# Regulation of the HBV Entry Receptor NTCP and its Potential in Hepatitis B Treatment

**DOI:** 10.3389/fmolb.2022.879817

**Published:** 2022-04-12

**Authors:** Yan Li, Jun Zhou, Tianliang Li

**Affiliations:** Key Laboratory of Animal Resistance Biology of Shandong Province, Institute of Biomedical Sciences, College of Life Sciences, Shandong Normal University, Jinan, China

**Keywords:** NTCP, HBV, transcriptional regulation, post-translational regulation, HBV entry

## Abstract

Hepatitis B virus (HBV) is a globally prevalent human DNA virus responsible for more than 250 million cases of chronic liver infection, a condition that can lead to liver inflammation, cirrhosis, and hepatocellular carcinoma. Sodium taurocholate co-transporting polypeptide (NTCP), a transmembrane protein highly expressed in human hepatocytes and a mediator of bile acid transport, has been identified as the receptor responsible for the cellular entry of both HBV and its satellite, hepatitis delta virus (HDV). This has led to significant advances in our understanding of the HBV life cycle, especially the early steps of infection. HepG2-NTCP cells and human NTCP-expressing transgenic mice have been employed as the primary cell culture and animal models, respectively, for the study of HBV, and represent valuable approaches for investigating its basic biology and developing treatments for infection. However, the mechanisms involved in the regulation of NTCP transcription, translation, post-translational modification, and transport are still largely elusive. Improvements in our understanding of NTCP biology would likely facilitate the design of new therapeutic drugs for the prevention of the *de novo* infection of naïve hepatocytes. In this review, we provide critical findings regarding NTCP biology and discuss important questions that remain unanswered.

## 1 Introduction

Hepatitis B virus (HBV) infection represents a major public healthcare challenge globally. The World Health Organization (WHO) has estimated that 296 million people were living with chronic hepatitis B infection in 2019, resulting in an estimated 820,000 deaths, with 1.5 million new infections being diagnosed each year (https://www.who.int/news-room/fact-sheets/detail/hepatitis-b). In a multi-center international study involving 161 countries, the global prevalence of HBV surface antigen (HBsAg) was estimated to be 3.61%, with the highest rates being detected in Africa (8.83%) and Western Pacific regions (5.26%) (Schweitzer et al., 2015). Although vaccination has proven successful, high viral turnover rates have resulted in vaccine-related escape mutants, rendering HBV infection a serious problem (Shepard et al., 2005). HBV infection can become chronic and eventually lead to end-stage liver disease and/or the development of hepatocellular carcinoma (HCC) (Ringehan et al., 2017). Owing to the high prevalence of HBsAg, the WHO is working on improving prevention, diagnostic, and treatment strategies to help countries achieve the global hepatitis elimination targets set under the Sustainable Development Agenda 2030 (https://www.who.int/news-room/fact-sheets/detail/hepatitis-b). Over the last 5 years, attention has increasingly focused on the important topics of HBV screening, diagnosis of HBV infection, and appropriate linkage to care. There have also been rapid clinical developments toward a functional cure of HBV infection, and novel compounds are currently in various phases of development (Nguyen et al., 2020). Despite some advances, issues with screening, diagnosis, and treatment of HBV infection remain (Almeida et al., 2021).

Sodium taurocholate co-transporting polypeptide (NTCP) is a sodium-dependent uptake transporter residing in the basolateral membrane of hepatocytes and is involved in the hepatic uptake of conjugated bile salts (Watashi et al., 2014a). In addition to its transport function, NTCP has been identified as an entry receptor for HBV and its satellite, hepatitis delta virus (HDV). The latter is dependent on HBV-encoded proteins for envelopment (Yan et al., 2012). This finding led to the development of reliable cell culture systems that allowed a better understanding of the molecular mechanisms of entry during the early viral lifecycle (Rybicka and Bielawski 2020). In general, the viral entry step is an attractive target for the development of antiviral agents. Pharmacological studies suggest that NTCP can serve as a therapeutic target, which has led to a surge in research focusing on NTCP as a drug target to inhibit HBV infections. Mainly focusing on recent findings, this review summarizes the current knowledge of the molecular mechanisms related to the HBV entry receptor NTCP and discusses its implications.

## 2 The Role of Sodium Taurocholate Co-Transporting Polypeptide in the Enterohepatic Circulation of Bile Acids

The human NTCP-encoding gene—solute carrier family 10 member 1 (SLC10A1)—was initially cloned by Hagenbuch and Meier in 1994 (Hagenbuch and Meier 1994), and was the first NTCP-related gene to be identified. Subsequently, Hagenbuch et al. (1996) showed that Xenopus oocytes injected with total mRNA extracted from rat hepatocytes exhibited Na^+^-dependent bile acid uptake activity, and this activity was inhibited by targeting NTCP transcripts using antisense oligonucleotides. These elegant experiments provided compelling evidence that NTCP represents the major route for Na^+^-dependent uptake of bile acids, at least in the rat liver (Alrefai and Gill 2007). NTCP belongs to the SLC10 transporter family of proteins, which consists of seven members, namely, NTCP; apical sodium-dependent bile acid transporter (ASBT, encoded by SLC10A2); sodium-dependent organic anion transporter (SOAT; SLC10A6); P3/SLC10A3 (encoded by SLC10A3); P4/SLC10A4 (SLC10A4); P5/SLC10A5 (SLC10A5); and SLC10A7 (SLC10A7). The SLC10 family is mainly responsible for transporting physiological substrates, such as bile salts, steroid hormones, and various drugs (Claro da Silva et al., 2013). NTCP is a transmembrane protein specifically found in the basolateral membrane of hepatocytes where it functions in the basolateral uptake of bile acids from the portal blood into hepatocytes (Lee et al., 2017).

Bile acids are synthesized from cholesterol in the liver and subsequently stored in the gallbladder. They are essential for the absorption of lipids, cholesterol, and lipid-soluble vitamins owing to their amphipathic nature and ability to form mixed micelles. Most of the bile acids released into the small intestine after a meal are reabsorbed by the intestinal wall and returned to the liver through the portal vein for resecretion (Ticho et al., 2019). Bile acids from sinusoidal blood enter the canaliculus through hepatocytes and are taken up by hepatocytes via NTCP (Zhao et al., 2021). NTCP co-transports bile acids at a Na^+^/taurocholate stoichiometry of 2:1, and is responsible for the uptake of >80% of conjugated taurocholate and <50% of unconjugated cholate from the blood into hepatocytes. Meanwhile, the bile salt export pump (BSEP), which belongs to the ATP-binding cassette transporter family, is primarily found in the canalicular membrane of hepatocytes, and effluxes bile salts from hepatocytes into the bile. Hence, NTCP and BSEP are important proteins for the transportation of bile acids and the maintenance of bile acid homeostasis (Alrefai and Gill 2007; Lu et al., 2019). In addition to basic physiological substrates, such as glycine- and taurine-conjugated bile acids and sulfated and unconjugated bile salts, NTCP also transports estrone-3-sulfate [E (1)S], dehydroepiandrosterone sulfate (DHEAS), bromosulfophthalein, thyroid hormones, and even exogenous chemicals (Petzinger 1994; Mita et al., 2006; Visser et al., 2010; Stieger 2011).

A decrease in NTCP functional activity is linked to impaired bile salt homeostasis and can lead to cholestasis (Stieger 2011). Cholestasis is caused by a reduction of bile flow and results in the retention of bile salts within hepatocytes. Owing to their detergent properties, the accumulated bile salts can become cytotoxic to hepatocytes, including mitochondrial damage, finally leading to liver injury (Sokol et al., 2006). Research has shown that a marked reduction in NTCP mRNA levels contributes to inflammation-induced cholestasis in humans (Zollner et al., 2001), and that NTCP expression can be downregulated in isolated and cultured human hepatocytes following treatment with interleukin 6 (IL-6), IL-1β, or tumor necrosis factor-alpha (TNF-α) (Le Vee et al., 2008; Le Vee et al., 2009). NTCP was downregulated at the level of protein, but not mRNA, in liver samples obtained from patients with progressive familial intrahepatic cholestasis, indicating a post-translational regulation of NTCP in this disease (Keitel et al., 2005). Additionally, a novel disorder called NTCP deficiency, which is an inborn error of bile acid metabolism caused by biallelic mutations in the SLC10A1 gene, was first described by Vaz et al., in 2015 (Vaz et al., 2015). Subsequently, an increasing number of patients have been diagnosed with this condition, many of whom presented no manifestations other than hypercholanemia. NTCP deficiency was shown to be ethnicity-dependent, with the functionally impairing c.800C > T (pSer267Phe) variant being found in Chinese Americans (7.5%) and the c.668T > C (pIle223Thr) variant in African Americans (5.5%) and Hispanic Americans (0.55%) (Claro da Silva et al., 2013). Further molecular and clinical evidence is needed to provide comprehensive insight into the mechanisms underlying the pivotal role of NTCP in these disorders, and allow the development of therapeutic strategies aimed at their treatment.

## 3 The Role of Sodium Taurocholate Co-Transporting Polypeptide in Hepatitis B Virus Entry

### 3.1 Identification of Sodium Taurocholate Co-Transporting Polypeptide as a Receptor for Hepatitis B Virus

HBV, a causative agent for acute/chronic hepatitis and HCC, is transmitted through contact with infected blood or other body fluids and triggers immune-mediated liver diseases of varying severity and duration. HBV has a circular, partly double-stranded, enveloped DNA genome that selectively enters hepatocytes where it delivers its genome, thereby initiating a multifaceted process of viral replication (Iannacone and Guidotti 2022). The HBV envelope contains three forms of the HBV surface protein (HBsAg), namely the large (L), middle (M), and small (S) proteins. Importantly, the preS1 domain of the large envelope protein has been identified as an essential structure for HBV attachment and entry (Sureau et al., 1993). However, the functional receptor mediating HBV entry into hepatocytes remained unknown for more than 20 years since the discovery of the virus, until NTCP was identified as being critical for preS1 binding and HBV infection (Yan et al., 2012).

**FIGURE 1:**
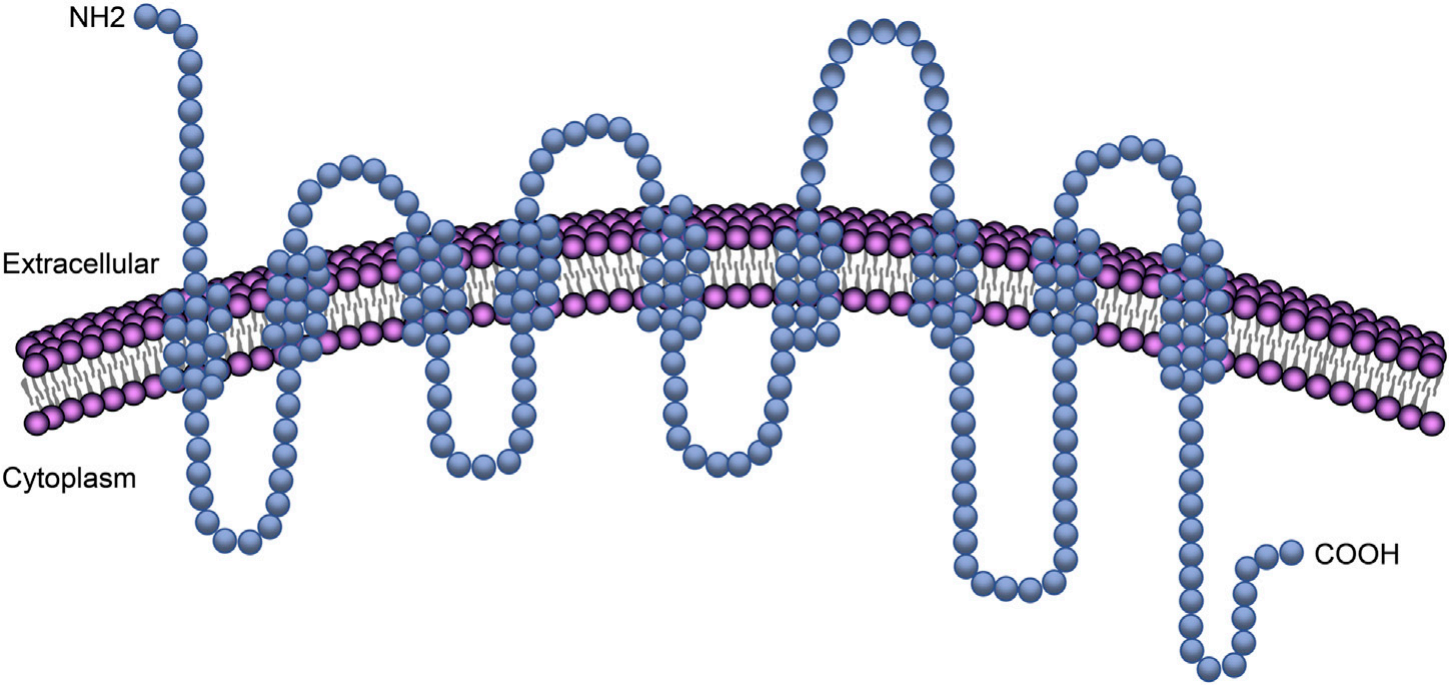
NTCP transmembrane domains ​in the plasma membrane. The transmembrane protein NTCP has a putative nine transmembrane domains with a topology predicted to consist of an extracellular N-terminus and an intracellular C-terminus.

NTCP is located on the basolateral membrane domain (blood-side) of hepatocytes and is broadly expressed in humans, rats, and monkeys, among other species (Qiu et al., 2013). NTCP is expressed exclusively in the liver. Human NTCP (hNTCP, encoded by the SLC10A1 gene) is a protein of 349 amino acids with an apparent mass of 56 kDa (Dawson et al., 2009; Watashi and Wakita 2015). Although the crystal structure of hNTCP has not been solved, a series of modeling, mutagenesis, and biochemical analyses suggest that this protein has a putative nine transmembrane domains with a topology predicted to consist of an extracellular N-terminus and an intracellular C-terminus (Hu et al., 2011; Fukano et al., 2019; Appelman et al., 2021) (Figure 1). Which region(s) of NTCP is essential for viral attachment and the subsequent triggering of internalization remains incompletely understood. Current evidence suggests that hNTCP residues 157 to 165 (KGIVISLVL) are important for preS1 binding and, thus, HBV infection (Yan et al., 2012; Ni et al., 2014). By analyzing the susceptibility of NTCP variants to HBV attachment, Müller et al. (2018) showed that a single amino acid at position 158 of NTCP is critical for HBV binding and subsequent infection. Similarly, Junko and others demonstrated that the sequence of amino acid 158 was a determining factor for the attachment of the HBV envelope protein to the host cell (Takeuchi et al., 2019). In addition, it was found that the molecular determinants for the transporter function of NTCP overlapped with those for its ability to support HBV entry (Yan et al., 2014). Mutations in the amino acids of NTCP that are critical for bile salt binding (N262A, Q293A/L294A) abrogated both the binding of NTCP to the preS1 peptide and infection by HBV. The S267F variant of NTCP could neither bind to the preS1 region nor support HBV infection in cell culture, thereby supporting the role of NTCP as the cellular receptor for HBV infection in humans (Peng et al., 2015; Lee et al., 2017; An et al., 2018). A recent study demonstrated that hepatocytes expressing the NTCP-S267F variant, introduced into the SLC10A site via CRISPR editing, are resistant to HBV infection (Uchida et al., 2021). However, patients homozygous for S267F can still be infected by HBV, suggesting the existence of alternative receptors that allow viral entry in the absence of functional NTCP (Hu et al., 2016).

HBV primarily infects humans, chimpanzees, and several other great apes, but not monkeys or lower primates. Viral infections in non-susceptible species are known to be mainly restricted at the entry level as viral replication can be achieved in cells from the relevant species when the cells are transfected with the viral genome (Yan et al., 2013). Lempp and others reported that hepatocytes from cynomolgus macaques, rhesus macaques, and pigs expressing hNTCP become fully susceptible to HBV with efficiencies comparable to that for human hepatocytes. However, when expressed in rodent or canine hepatocytes, hNTCP can support HBV attachment but not the establishment of HBV infection (Lempp et al., 2017). Moreover, the substitution of residues 85–87 of murine NTCP (mNTCP) with those of human NTCP is sufficient to facilitate HBV entry into the cell but not to support infection (Yan et al., 2013). These observations imply that other host factors besides NTCP are required for HBV internalization in hepatocytes of these animals and that the differences between species, such as NTCP structure and (co)receptors, affect HBV susceptibility and infection efficiency.

### 3.2 Sodium Taurocholate Co-Transporting Polypeptide Mediates Hepatitis B Virus Infection

HBV infection into host hepatocytes involves multiple steps. In the initiation of infection, HBV reversibly attaches to heparan sulfate proteoglycans on the cell surface via a highly conformational determinant region 1) of the HBsAg glycoprotein (Sureau and Salisse 2013). HBV subsequently interacts with its specific receptor with high affinity, thereby triggering viral internalization. After endocytosis-mediated internalization, the virus fuses with the cellular membrane compartment. However, the precise mechanisms are not yet fully understood (Watashi et al., 2014a).

Data collected to date clearly indicate that NTCP can serve as an HBV receptor, especially *in vitro* (Li and Tong 2015). NTCP mediates the specific binding of HBV to the host cell surface with high affinity by interacting with the preS1 region of the large surface protein (LHB) of HBV. NTCP confers HBV susceptibility to human hepatic cell lines such as HepG2, Huh7, or undifferentiated HepaRG cells, which are originally non-susceptible to infection (Iwamoto et al., 2014; Ni et al., 2014). This easily manipulated model of cell infection has been used to decipher the early steps of HBV entry. However, the expression of NTCP alone is not sufficient for efficient HBV internalization into hepatocytes, and additional host factors are likely to be required for susceptibility to HBV infection, potentially through the formation of a complex and a multistep entry process. For example, host epidermal growth factor receptor (EGFR) was reported to interact with NTCP and mediate HBV internalization (Iwamoto et al., 2019), a finding that potentially accounts for the low rate of infection in the HepG2 cell line, in which EGFR expression is undetectable (Zhao et al., 2013). Kinesin family member 4 (KIF4) regulates the levels of surface NTCP via the anterograde transport of NTCP to the cell surface, potentially also mediating HBV entry (Gad et al., 2021).

## 4 Regulation of Sodium Taurocholate Co-Transporting Polypeptide Expression

The human, rat, and mouse SLC10A1/Slc10a1 genes span 21.4, 13.6, and 12.5 kb and map to chromosomes 14q24, 6q24, and 12D1, respectively. The human SLC10A1 gene comprises five exons and contains an open reading frame of 1,047 bp encoding a 349-amino acid protein with a calculated molecular mass of approximately 38 kDa (Döring et al., 2012). NTCP is located predominantly on the basolateral membrane of hepatocytes and is responsible for the uptake of conjugated bile acids from the blood into the liver (Anwer and Stieger 2014). Nevertheless, elevated bile salt levels in hepatocytes can inhibit NTCP expression at both the transcriptional and post-translational levels through a negative feedback loop mediated by diverse cellular signaling pathways.

### 4.1 Transcriptional Regulation of Sodium Taurocholate Co-Transporting Polypeptide

**FIGURE 2:**
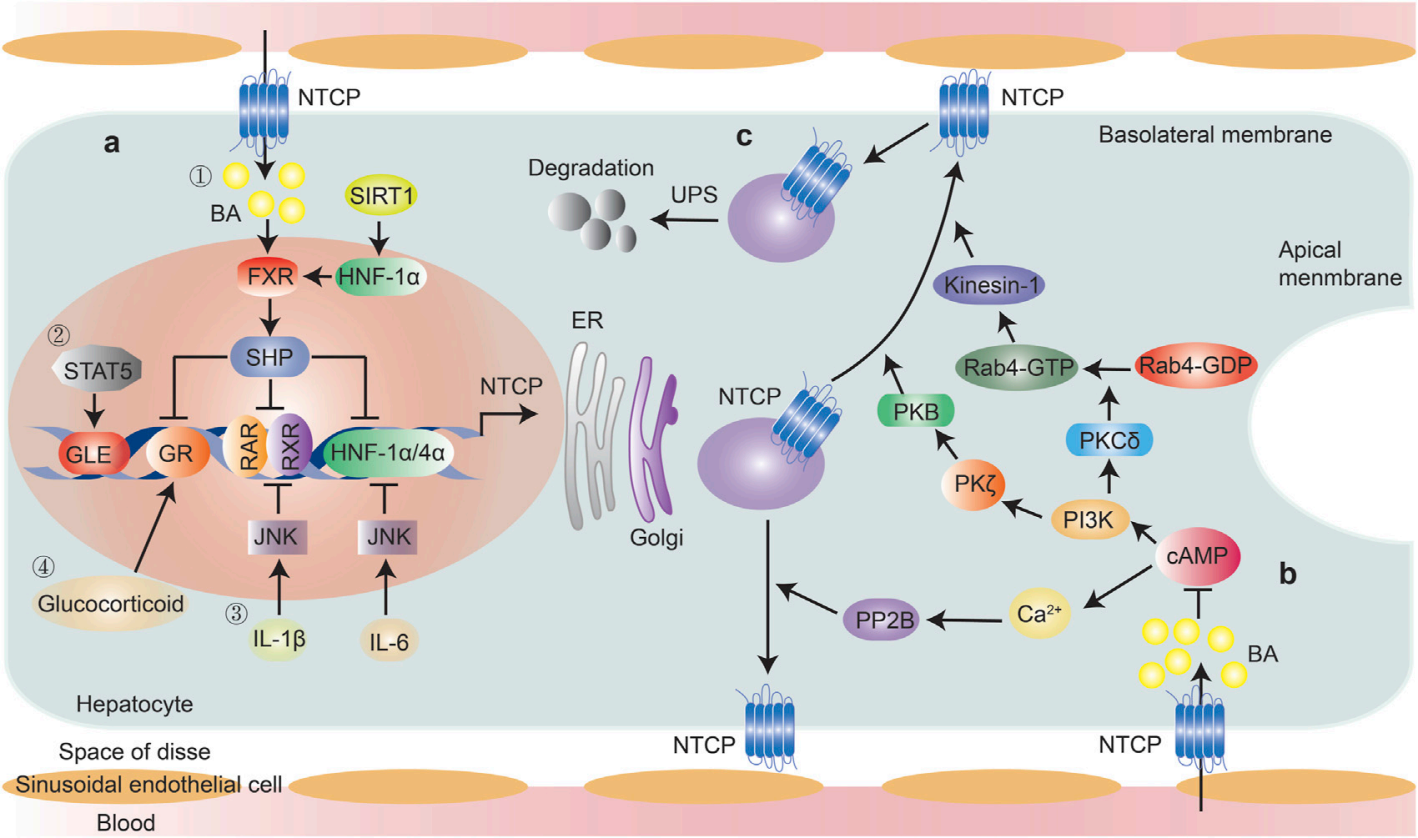
Regulation of NTCP expression. Transcriptional and post-translational regulation of NTCP were summarized. **(A)** Transcriptional regulation, ① Bile acid-induced, FXR-mediated induction of the nuclear repressor SHP is a key mechanism reducing NTCP expression, through its interference with the RXR-RAR heterodimer, HNF-1α and HNF-4α, which have binding sites within the NTCP promoter. FXR expression can be modulated by SIRT1 through HNF-1α; ② STAT5 directly bound to NTCP promoter and mediates NTCP expression; ③ IL-6 down-regulates the expression of NTCP through suppression of HNF1α and HNF4α in JNK pathway-dependent manner, IL-1β down-regulates the expression of NTCP via suppression of the RAR/RXR complex in JNK pathway-dependent manner; ④ Glucocorticoid increases NTCP expression in a GR-dependent manner, which was inhibited by FXR-induced expression of SHP. **(B)** Post-translational regulation, PP2B and PI3K/PKB/PKC axis facilitate the intracellular movement of NTCP towards the plasma membrane following cAMP activation. Elevated bile acid levels inhibit cAMP activation; **(C)**. NTCP protein abundance was controlled by ubiquitin-proteasome system. Abbreviations: BA, bile acid; FXR, farnesoid X receptor; SHP, small heterodimer partner; HNF-1α, hepatocyte nuclear factor 1 alpha; SIRT1, hepatic sirtuin 1; STAT5, signal transducer and activator of transcription 5; GLE, interferon-gamma (IFN-γ)-activated sequence-like element; GR, glucocorticoid receptor; RXR, retinoid X receptor; RAR, RXR-retinoic acid receptor; PP2B, protein phosphatase 2B; UPS, ubiquitin-proteasome system.

Most studies on NTCP transcriptional regulation are based on rodent models and engineered HepG2-NTCP or other cells because NTCP shows minimal endogenous expression in hepatocellular cell models and is rapidly lost from primary human hepatocytes following their isolation (Xia et al., 2017). Key transcriptional regulators of NTCP identified to date include farnesoid X receptor (FXR)/small heterodimer partner (SHP), the homeodomain protein hepatocyte nuclear factor 1 alpha (HNF-1α), HNF-4α, retinoid X receptor-alpha (RXRα), HNF-3β, and signal transducer and activator of transcription 5 (STAT5) (Ganguly et al., 1997; Lu et al., 2019). Other factors, such as proinflammatory cytokines, hormones, and several chemical compounds, are also suggested to participate in the transcriptional regulation of NTCP (Figure 2).

NTCP mRNA regulation is mainly linked to bile acid concentration, which serves as an adaptive response to block excessive bile acid accumulation in hepatocytes under pathophysiological conditions (Xia et al., 2017). NTCP is downregulated during cholestasis. Bile acid-induced, FXR-mediated induction of the nuclear repressor SHP has been proposed to be a key mechanism underlying the reduction in NTCP expression through interfering with the RXR-retinoic acid receptor (RAR) heterodimer, for which there is a binding site within the NTCP promoter (Anwer 2004). FXR is a bile acid-activated nuclear receptor (BAR) and is mainly expressed in the liver and intestine. In the liver, NTCP was reported to be strongly repressed in wild-type (WT) mice fed a colic acid-containing diet. This repression was largely retained in SHP-null mice, in agreement with the proposed role of SHP in the negative regulation of NTCP expression. This phenomenon also indicated that SHP-independent mechanisms may be involved in mediating NTCP repression in cholestasis (Wang et al., 2003). In the distal ileum, FXR activation also instigates the production of fibroblast growth factor 19 (FGF19; FGF15 in mice), which then reaches hepatocytes via the portal circulation and activates the fibroblast growth factor receptor 4 (FGFR4)/β-Klotho complex, thereby triggering intracellular signaling pathways in the liver to maintain the bile acid balance. The administration of recombinant hFGF19 downregulates NTCP mRNA by ∼50% in WT mice, indicating that FXR further modulates NTCP mRNA expression via FGF15/19 signaling (Inagaki et al., 2005; Slijepcevic et al., 2017). In addition, the glucocorticoid receptor (GR), a ligand-activated transcription factor, can directly bind and activate the NTCP promoter. Human NTCP expression is upregulated by the GR ligand dexamethasone in Huh-7 cells and augmented by peroxisome proliferator-activated receptor-γ coactivator-1α (PGC-1α), while GR-mediated activation is inhibited by SHP downstream of FXR (Eloranta et al., 2006). Furthermore, it was reported that FXR expression is regulated by hepatic sirtuin 1 (SIRT1), a NAD-dependent deacetylase that has been found to couple the deacetylation of many transcription factors and cofactors, such as NF-κB, PGC-1α and FXR, to NAD^+^ hydrolysis (Yu et al., 2016).

HNF-1α and HNF-3β are also essential regulators of bile acid metabolism. HNF-1α can bind and transactivate the rat Ntcp promoter, but not that of humans or mice, whereas HNF-3β mediates the transcriptional repression of the NTCP/Ntcp promoter in all three species via directly binding to its response elements (Jung et al., 2004; Zhao et al., 2021). HNF-1α^−/−^ livers exhibit decreased NTCP expression in the basolateral membrane, leading to impaired portal bile acid uptake and elevated plasma bile acid concentrations (Yu et al., 2016). HNF-1α also regulates the expression of the nuclear receptor FXR, a key regulator of NTCP expression (Shih et al., 2001). As mentioned above, SIRT1 modulates FXR transcriptional regulation through HNF-1α, and SIRT1 deficiency in the liver was found to decrease the binding of HNF-1α to the FXR promoter and thus reduce its expression, resulting in impaired bile acid metabolism (Purushotham et al., 2012). HNF-4α directly binds to the mouse Ntcp promoter through functional HNF-4α response elements while PGC-1α potentiates HNF-4α-induced NTCP transactivation (Geier et al., 2008). Importantly, as HNF-1α was shown to control HNF-4α transcription, repressed NTCP expression in HNF-1α deficiency may potentially reflect an HNF-4α-mediated mechanism (Odom et al., 2004).

STAT5 was reported to directly bind to interferon-gamma (IFN-γ)-activated sequence-like elements (GLEs) in the Ntcp promoter as well as mediate the upregulation of Ntcp expression by prolactin in the rat (Ganguly et al., 1997). Notably, however, Ntcp expression was found to be reduced in pregnant rats in late gestation despite the increase in the level of STAT5. This suggests that a STAT5-independent pathway that is activated during pregnancy exerts a greater effect than STAT5 on Ntcp expression (Zhao et al., 2021). Bu and others investigated the impact of STAT5 signaling on the expression of the mouse and human Ntcp/NTCP gene using berberine, an inhibitor of STAT signaling. Through assessing the binding of phospho-STAT5 protein to STAT5 response elements in the NTCP promoter, the authors determined that STAT5 regulates the expression of both mouse and human Ntcp/NTCP (Bu et al., 2017), as previously also reported for the rat.

Cholestasis can trigger the release of cytokines such as IL-6, IL-1β, and TNF-α, which, in turn, repress NTCP transcription. IL-6 is an important cytokine with roles in inflammation and liver regeneration, and NTCP expression is known to be strongly regulated by IL-6. A decrease of up to 98% in NTCP mRNA steady-state levels was detected in primary human hepatocyte (PHH) and HepaRG cells after IL-6 treatment (Bouezzedine et al., 2015). The effect of IL-6 on NTCP has also been investigated in mice through the injection of turpentine, which induces an acute phase response with IL-6 production. Turpentine treatment resulted in the downregulation of NTCP mRNA expression in WT mice, but not in IL-6 KO animals (Siewert et al., 2004). In addition, Yan et al. (2019) found that there was a sharp rise in IL-6 mRNA levels in mice injected with LPS, an effect that was accompanied by a reduction in NTCP transcript levels. However, NTCP mRNA levels showed a partial recovery when serum IL-6 was neutralized with IL-6 antibodies, indicating that IL-6 can downregulate the expression of NTCP in inflammation at the transcriptional level. Mechanistic studies indicated that the downregulation of NTCP could be mediated by the activation of c-Jun N-terminal kinase (JNK), which suppresses the expression and binding activity of multiple nuclear factors such as HNF-1α and HNF-4α (Geier et al., 2007). Similarly, a marked downregulation of NTCP mRNA expression was detected in HepaRG cells in response to 24 h of exposure to IL-1β at the concentration of 1 ng/ml (Le Vee et al., 2008). Moreover, this effect was shown to be mediated via the suppression of the RAR/RXR complex in a JNK pathway-dependent manner, resulting in the downregulation of NTCP promoter activity and transport activity (Li et al., 2002). The effects of IL-1β and TNFα have been investigated in rats, and the inactivation of either cytokine was found to prevent the downregulation of NTCP mRNA expression. In contrast to the predominant role of IL-1β in a complex signaling network involved in NTCP regulation in cholestatic liver injury, TNF-α represents the master cytokine responsible for the HNF1-dependent reduction of NTCP levels in CCl4-induced toxic liver injury (Geier et al., 2003). Another cytokine within the IL-6 family, oncostatin M (OSM), also dose-dependently induced the downregulation of NTCP expression, an effect that was highly correlated with that of IL-6 (Le Vee et al., 2011).

Hormones such as estrogen, adrenal glucocorticoid, prolactin, growth hormone, and thyroid hormone are also suggested to participate in the regulation of NTCP expression (Simon et al., 2004; Cheng et al., 2007; Rose et al., 2011). For example, Rose et al. (2011) demonstrated that *ex vivo* glucocorticoid treatment increases NTCP expression in a GR-dependent manner in both the human and mouse liver. Meanwhile, chemical compounds such as dioxin, rifampicin, cholestyramine, phenobarbital, and oltipraz have also been shown to participate in the regulation of NTCP expression (Cheng et al., 2007; Zhao et al., 2021). However, the mechanisms underlying the effects of these hormones and compounds on NTCP expression need further investigation.

### 4.2 Post-Translational Regulation of Sodium Taurocholate Co-Transporting Polypeptide

NTCP, an integral membrane glycoprotein, is localized to the plasma membrane and endocytic vesicles. The localization and membrane expression of NTCP is controlled by post-translational mechanisms involving phosphorylation or dephosphorylation, translocation to and retrieval from the plasma membrane, and degradation by the ubiquitin-proteasome system (UPS) (Anwer 2014). These post-translational regulatory mechanisms are mediated via signaling pathways involving cyclic adenosine monophosphate (cAMP), calcium, phosphoinositide-3-kinase (PI3K), protein kinase C (PKC), nitric oxide, and protein phosphatases (Anwer and Stieger 2014). For example, cAMP stimulates the dephosphorylation and consequent translocation of NTCP to the plasma membrane by dephosphorylating the Ser-226 site in the third cytoplasmic loop of NTCP (Anwer et al., 2005). Moreover, cAMP can enhance taurocholate uptake by promoting the insertion of NTCP into the plasma membrane via increases in intracellular Ca^2+^ concentrations and the subsequent activation of protein phosphatase 2B (PP2B) via Ca^2+^-calmodulin kinase (Mukhopadhayay et al., 1997; Anwer and Stieger 2014).

The PI3K-signaling pathway, which comprises three downstream effectors (protein kinase B [PKB/AKT]), P70 S6 kinase [p70^S6K^], and PKC) plays a role in the cAMP-mediated translocation of NTCP. The effect of cAMP is not mediated via p70^S6K^ because treatment with rapamycin, an inhibitor of p70^S6K^, does not inhibit cAMP-mediated stimulation of Na^+^-taurocholate uptake (Webster and Anwer 1999; Webster et al., 2000). Transfection with constitutively active PKB was shown to increase PKB activity, taurocholate uptake, and NTCP translocation, whereas inhibiting PKB blocked cell swelling- and cAMP-induced increases in taurocholate uptake and NTCP translocation (Webster et al., 2002). PKC is expressed as multiple isoforms, among which PKCδ and PKCζ have been suggested to play a role in the cAMP-induced translocation of NTCP to the cell surface. The PKCδ isoform mediates cAMP-induced translocation of NTCP via the activation of Rab4, which is associated with early endosomes and is involved in vesicular trafficking. These effects of PKCδ are mediated by its plasma membrane localization rather than its kinase activity (Park et al., 2012). Moreover, PKCζ activation enhances the cAMP-promoted motility of NTCP-containing vesicles, while specific inhibition of PKCζ exerts the opposite effect, indicating that PKCζ is specifically required for the cAMP-induced intracellular movement of vesicles that contain the NTCP transporter (Sarkar et al., 2006).

In addition, maturing NTCP is degraded by the ubiquitin-proteasome system at the level of ER-associated degradation (ERAD), and an imbalance in NTCP synthesis and degradation can result in intracellular NTCP deposits and subsequently lead to cholestasis (Kühlkamp et al., 2005). In summary, PP2B and PI3K/PKB/PKC axis facilitate the intracellular movement of NTCP toward the plasma membrane following cAMP activation, while the ubiquitin-proteasome system controls NTCP protein abundance (Figure 2).

## 5 Sodium Taurocholate Co-Transporting Polypeptide as a Target for Anti-Hepatitis B Virus Agents

As the first step in viral infection, viral entry represents an attractive target for the development of antiviral agents. Using entry inhibitors, HBV infection can be blocked before the virus produces its genomic material or alters infected cells. The inhibition of HBV entry is effective at preventing *de novo* infection during post-exposure prophylaxis, organ transplantation, reactivation following therapeutic immunosuppression, or perinatal transfer from an infected mother to her children (Urban et al., 2014). The identification of NTCP as a cellular receptor for HBV, involving a specific interaction between NTCP and the preS1 domain of the HBV large envelope protein, has permitted the rational design of drugs targeting viral entry (Trépo et al., 2014). Many types of HBV entry inhibitors targeting NTCP-mediated viral infection have been identified to date, including bile acids and their derivatives, peptides, chemical compounds, and neutralizing antibodies (Table 1).

**TABLE 1:** Examples of HBV entry inhibitors targeting NTCP-mediated viral infection.

Drugs	Mechanism	References
**Bile Acids and Their Derivatives**		
Bile acids: taurocholate, tauroursodeoxycholate, bromosulfophthalein	Competitively inhibit NTCP-mediated HBV entry	Yan et al. (2014), Zhao et al. (2021)
Bile acid derivatives: OCA, INT-767	FXR agonist, blocks HBV entry by inhibiting NTCP	Ito et al. (2021)
	Dual agonist of FXR and TGR5, blocks HBV entry by inhibiting NTCP	
DBADs (DBA-41)	Binds to NTCP	Liu et al. (2021)
**Peptides**		
Myrcludex B	Blocks the NTCP receptor	Volz et al. (2013), Bogomolov et al. (2016), Cheng et al. (2021)
Cyclosporin A and its derivatives	Block the NTCP receptor	Watashi et al. (2014b), Nkongolo et al. (2014), Shimura et al. (2017)
Macrocyclic peptides: WD1, WL2, and WL4	Interact with NTCP, without inhibiting the transporter activity of NTCP	Passioura et al. (2018)
**Chemical compounds**		
EGCG	Down-regulates NTCP protein	Huang et al. (2014)
Irbesartan	Inhibits NTCP via targeting SLC10A1	Ko et al. (2015)
Ezetimibe	Inhibits myr-preS1 peptide binding	Bays et al. (2008), Blanchet et al. (2014); König et al. (2014)
Fasiglifam	Inhibits NTCP	Nio et al. (2018)
Proanthocyanidin and its analogs	Directly targets the preS1 region of LHB	Tsukuda et al. (2017)
Vanitaracin A	Directly interacts with NTCP	Kaneko et al. (2015)
NTI-007	Tightly binds to NTCP and induces autophagy	Zhang et al. (2015)
Ritonavir	Interrupts NTCP function	Blanchet et al. (2014)
Ro41-5,253	Represses the NTCP promoter by antagonizing RAR	Tsukuda et al. (2015)
Evans blue	Inhibits the binding of preS1 to NTCP	Xiao et al. (2019)
NPD8716	Interacts with NTCP	Kaneko et al. (2018)
**Neutralizing antibodys**		
N6HB426-20	Anti-NTCP	Takemori et al. (2022)
MA18/7	Anti-preS1	Glebe et al. (2003)
2H5-A14	Anti-preS1	Li et al. (2017)

NTCP substrates such as taurocholate, tauroursodeoxycholate, and bromosulfophthalein competitively inhibit NTCP-mediated HBV entry because the molecular determinants for HBV entry overlap with those for bile salt uptake by NTCP (Yan et al., 2014; Zhao et al., 2021). The bile acid derivatives obeticholic acid (OCA) and 6α-ethyl-24-nor-5β-cholane-3α,7α,23-triol-23 sulfate sodium salt (INT-767; a dual agonist of FXR and Takeda G protein-coupled receptor [TGR5]) can block HBV entry by inhibiting NTCP (Ito et al., 2021). Accordingly, bile acids and their derivatives hold potential for development into novel therapeutic drugs targeting HBV infection.

Myrcludex B is a linear, synthetic N-acetylated lipopeptide consisting of 47 amino acids of the preS1 region of the large surface antigen generated through solid-phase synthesis. It is the first entry inhibitor capable of inactivating NTCP *in vitro* and *in vivo*, and does so by interacting with HBV particles and competing for NTCP receptors. The concentration that blocks HBV and HDV entry is 100-fold lower than that required to inhibit bile acid transport by NTCP (Volz et al., 2013; Bogomolov et al., 2016; Cheng et al., 2021). At present, Myrcludex B has been approved in Europe for the treatment of patients with chronic HDV infection under the trade name Hepcludex^®^ (Takemori et al., 2022). It has been reported that cyclosporin A (CsA), another peptide inhibitor that is usually used as an immunosuppressant in organ transplantation, as well as its analogs, can inhibit HBV infection by targeting NTCP and interfering with the interaction between preS1 and NTCP, thereby blocking HBV entry into hepatocytes (Watashi et al., 2014b; Nkongolo et al., 2014). Some macrocyclic peptides (WD1, WL2, and WL4) were also found to interact with NTCP, thereby inhibiting HBV infection, but with NTCP-mediated bile acid uptake (Passioura et al., 2018).

Regarding chemical compounds, numerous drugs have been developed to block the interaction between HBV and NTCP. Drugs such as irbesartan, ezetimibe, and ritonavir were identified as HBV inhibitors based on their potent inhibitory effect on NTCP metabolic function (Bays et al., 2008; Blanchet et al., 2014; König et al., 2014; Ko et al., 2015). Moreover, compounds obtained from herbal medicines, such as epigallocatechin-3-gallate (EGCG) (Huang et al., 2014), a flavonoid belonging to the catechin subclass present in green tea, can mitigate HBV entry by accelerating NTCP degradation. Proanthocyanidin, an oligomeric flavonoid, inhibits HBV entry into host cells by targeting the HBV large surface protein, and does so with only limited cytotoxic effect (Tsukuda et al., 2017). Vanitaracin A, a tricyclic polyketide, directly interacts with NTCP and impairs its bile acid transport activity (Kaneko et al., 2015). Meanwhile, Evans blue can inhibit the binding of viral preS1 to host cells through NTCP as well as virus capsid assembly by targeting the host’s big potassium (BK) channels. Accordingly, this compound represents a promising therapeutic option for the treatment of HBV infection (Xiao et al., 2019).

Several monoclonal antibodies that recognize the preS1 region and can inhibit viral entry have been reported to date. For example, MA18/7 and 2H5-A14 target the preS1 region and neutralize HBV infection (Glebe et al., 2003; Li et al., 2017). In addition, a monoclonal antibody against NTCP—N6HB426-20—has been shown to recognize the extracellular domain of human NTCP (residues 276/277 at the tip of extracellular loop-4 of NTCP) and block HBV entry into human liver cells *in vitro*, while exerting substantially less of an inhibitory effect on bile acid uptake (Takemori et al., 2022). With further improvements, this antibody may be a promising treatment option for patients with chronic hepatitis B.

## 6 Concluding Remarks

Chronic hepatitis remains a major global public health problem due to the risk of progression to chronic hepatitis, cirrhosis, and HCC. The discovery that NTCP, the key bile acid transporter expressed by liver cells, plays a crucial role in HBV entry into hepatocytes has significantly advanced our understanding of the HBV life cycle. This review has provided an overview of the complex regulation of NTCP activity and plasma membrane abundance, both of which are regulated at multiple levels. However, the individual contribution of each of these mechanisms under various conditions remains largely unclear, and further clarification will likely lead to the identification of additional anti-HBV drug candidates with novel modes of action.

The pharmacological inhibition of NTCP activity provides new avenues in the field of drug discovery as well as in the development of models for screening anti-HBV drugs. The advantage of NTCP-targeted HBV entry inhibitors is that they remain effective regardless of viral genotype, viral mutations, and the presence of subviral particles. Importantly, however, these agents also block NTCP-mediated bile acid transport into hepatocytes, and thus have the potential to cause adverse effects. Moreover, further *in vivo* data are required to allow the assessment of the efficiency and safety of NTCP-targeting drugs. Consequently, it is of great interest to identify specific viral entry inhibitors that do not affect the physiological function of NTCP. We speculate that LYTAC (lysosome-targeting chimeras) technology, which can trigger the targeted degradation of extracellular proteins and membrane-bound proteins by the proteasome, may be a good strategy for the downregulation of NTCP, although other mechanisms may be needed to ensure effective hepatic bile salt clearance in the case of excessive NTCP degradation by the proteasome. However, this hypothesis requires experimental validation.

In conclusion, NTCP is the most critical determinant of HBV entry into cells. However, the mechanisms involved in the regulation of NTCP remain largely elusive, the crystal structure of NTCP has yet to be elucidated, and many host factors involved in HBV infection have yet to be identified. Insight into these unknowns would be highly valuable for developing NTCP-targeting inhibitors and understanding the mechanism underlying HBV infection. Finally, bepirovirsen, an antisense oligonucleotide, was recently reported to inhibit HBV infection by targeting all HBV messenger RNAs. The results of a recent phase 2 randomized controlled trial of this drug showed that it performed with gratifying efficacy and a favorable safety profile (Yuen et al., 2021). Combining viral entry and replication inhibition will likely benefit the development of curative strategies for patients with chronic HBV.

## Abbreviations

OCA, obeticholic acid; INT‐767, 6α‐ethyl‐24‐nor‐5β‐cholane‐3α,7α,23‐triol-23, sulfate sodium salt; DBADs, Dimeric bile acid derivatives; TGR5, Takeda G protein-coupled receptor; EGCG, the green tea polyphenol epigallocatechin gallate; LHB, HBV, large surface protein; RAR, retinoic acid receptor.

## References

[B1] Almeida P. H. Matielo C. E. L. Curvelo L. A. Rocco R. A. Felga G. Della Guardia B. (2021). Update on the Management and Treatment of Viral Hepatitis. World J. Gastroenterol. 27 (23), 3249–3261. 10.3748/wjg.v27.i23.3249 34163109 PMC8218370

[B2] Alrefai W. A. Gill R. K. (2007). Bile Acid Transporters: Structure, Function, Regulation and Pathophysiological Implications. Pharm. Res. 24 (10), 1803–1823. 10.1007/s11095-007-9289-1 17404808

[B3] An P. Zeng Z. Winkler C. A. (2018). The Loss-Of-Function S267F Variant in HBV Receptor NTCP Reduces Human Risk for HBV Infection and Disease Progression. J. Infect. Dis. 218 (9), 1404–1410. 10.1093/infdis/jiy355 29905807 PMC6151084

[B4] Anwer M. S. Stieger B. (2014). Sodium-dependent Bile Salt Transporters of the SLC10A Transporter Family: More Than Solute Transporters. Pflugers Arch. Eur. J. Physiol. 466 (1), 77–89. 10.1007/s00424-013-1367-0 24196564 PMC3877701

[B5] Anwer M. S. Gillin H. Mukhopadhyay S. Balasubramaniyan N. Suchy F. J. Ananthanarayanan M. (2005). Dephosphorylation of Ser-226 Facilitates Plasma Membrane Retention of Ntcp. J. Biol. Chem. 280 (39), 33687–33692. 10.1074/jbc.M502151200 16027164

[B6] Anwer M. S. (2004). Cellular Regulation of Hepatic Bile Acid Transport in Health and Cholestasis. Hepatology 39 (3), 581–590. 10.1002/hep.20090 14999673

[B7] Anwer M. S. (2014). Role of Protein Kinase C Isoforms in Bile Formation and Cholestasis. Hepatology 60 (3), 1090–1097. 10.1002/hep.27088 24700589 PMC4141907

[B8] Appelman M. D. Wettengel J. M. Protzer U. Oude Elferink R. P. J. van de Graaf S. F. J. (2021). Molecular Regulation of the Hepatic Bile Acid Uptake Transporter and HBV Entry Receptor NTCP. Biochim. Biophys. Acta Mol. Cel Biol. Lipids 1866 (8), 158960. 10.1016/j.bbalip.2021.158960 33932583

[B9] Bays H. E. Neff D. Tomassini J. E. Tershakovec A. M. (2008). Ezetimibe: Cholesterol Lowering and beyond. Expert Rev. Cardiovasc. Ther. 6 (4), 447–470. 10.1586/14779072.6.4.447 18402536

[B10] Blanchet M. Sureau C. Labonté P. (2014). Use of FDA Approved Therapeutics with hNTCP Metabolic Inhibitory Properties to Impair the HDV Lifecycle. Antiviral Res. 106, 111–115. 10.1016/j.antiviral.2014.03.017 24717262

[B11] Bogomolov P. Alexandrov A. Voronkova N. Macievich M. Kokina K. Petrachenkova M. (2016). Treatment of Chronic Hepatitis D with the Entry Inhibitor Myrcludex B: First Results of a Phase Ib/IIa Study. J. Hepatol. 65 (3), 490–498. 10.1016/j.jhep.2016.04.016 27132170

[B12] Bouezzedine F. Fardel O. Gripon P. (2015). Interleukin 6 Inhibits HBV Entry through NTCP Down Regulation. Virology 481, 34–42. 10.1016/j.virol.2015.02.026 25765005

[B13] Bu P. Le Y. Zhang Y. Zhang Y. Cheng X. (2017). Berberine-induced Inactivation of Signal Transducer and Activator of Transcription 5 Signaling Promotes Male-specific Expression of a Bile Acid Uptake Transporter. J. Biol. Chem. 292 (11), 4602–4613. 10.1074/jbc.M116.757567 28154180 PMC5377776

[B14] Cheng X. Buckley D. Klaassen C. D. (2007). Regulation of Hepatic Bile Acid Transporters Ntcp and Bsep Expression. Biochem. Pharmacol. 74 (11), 1665–1676. 10.1016/j.bcp.2007.08.014 17897632 PMC2740811

[B15] Cheng D. Han B. Zhang W. Wu W. (2021). Clinical Effects of NTCP‐inhibitor Myrcludex B. J. Viral Hepat. 28 (6), 852–858. 10.1111/jvh.13490 33599010

[B16] Claro da Silva T. Polli J. E. Swaan P. W. (2013). The Solute Carrier Family 10 (SLC10): beyond Bile Acid Transport. Mol. Aspects Med. 34 (2-3), 252–269. 10.1016/j.mam.2012.07.004 23506869 PMC3602841

[B17] Dawson P. A. Lan T. Rao A. (2009). Bile Acid Transporters. J. Lipid Res. 50 (12), 2340–2357. 10.1194/jlr.R900012-JLR200 19498215 PMC2781307

[B18] Döring B. Lütteke T. Geyer J. Petzinger E. (2012). The SLC10 Carrier Family. Curr. Top. Membr. 70, 105–168. 10.1016/B978-0-12-394316-3.00004-1 23177985

[B19] Eloranta J. J. Jung D. Kullak-Ublick G. A. (2006). The Human Na+-Taurocholate Cotransporting Polypeptide Gene Is Activated by Glucocorticoid Receptor and Peroxisome Proliferator-Activated Receptor-γ Coactivator-1α, and Suppressed by Bile Acids via a Small Heterodimer Partner-dependent Mechanism. Mol. Endocrinol. 20 (1), 65–79. 10.1210/me.2005-0159 16123152

[B20] Fukano K. Tsukuda S. Watashi K. Wakita T. (2019). Concept of Viral Inhibitors via NTCP. Semin. Liver Dis. 39 (1), 078–085. 10.1055/s-0038-1676804 30809790

[B21] Gad S. A. Sugiyama M. Tsuge M. Wakae K. Fukano K. Oshima M. (2021). The Kinesin KIF4 Mediates HBV/HDV Entry through Regulation of Surface NTCP Localization and Can Be Targeted by RXR Agonists *In Vitro* . bioRxiv [Preprint]. 10.1101/2021.09.29.462331 PMC8970526 35312737

[B22] Ganguly T. C. O'Brien M. L. Karpen S. J. Hyde J. F. Suchy F. J. Vore M. (1997). Regulation of the Rat Liver Sodium-dependent Bile Acid Cotransporter Gene by Prolactin. Mediation of Transcriptional Activation by Stat5. J. Clin. Invest. 99 (12), 2906–2914. 10.1172/JCI119485 9185514 PMC508142

[B23] Geier A. Dietrich C. G. Voigt S. Kim S. K. Gerloff T. Kullak-Ublick G. A. (2003). Effects of Proinflammatory Cytokines on Rat Organic Anion Transporters during Toxic Liver Injury and Cholestasis. Hepatology 38 (2), 345–354. 10.1053/jhep.2003.50317 12883478

[B24] Geier A. Wagner M. Dietrich C. G. Trauner M. (2007). Principles of Hepatic Organic Anion Transporter Regulation during Cholestasis, Inflammation and Liver Regeneration. Biochim. Biophys. Acta Mol. Cel Res. 1773 (3), 283–308. 10.1016/j.bbamcr.2006.04.014 17291602

[B25] Geier A. Martin I. V. Dietrich C. G. Balasubramaniyan N. Strauch S. Suchy F. J. (2008). Hepatocyte Nuclear Factor-4α Is a central Transactivator of the Mouse Ntcp Gene. Am. J. Physiol. Gastrointestinal Liver Physiol. 295 (2), G226–G233. 10.1152/ajpgi.00012.2008 PMC2519858 18483185

[B26] Glebe D. Aliakbari M. Krass P. Knoop E. V. Valerius K. P. Gerlich W. H. (2003). Pre-s1 Antigen-dependent Infection of Tupaia Hepatocyte Cultures with Human Hepatitis B Virus. J. Virol. 77 (17), 9511–9521. 10.1128/jvi.77.17.9511-9521.2003 12915565 PMC187384

[B27] Hagenbuch B. Meier P. J. (1994). Molecular Cloning, Chromosomal Localization, and Functional Characterization of a Human Liver Na+/bile Acid Cotransporter. J. Clin. Invest. 93 (3), 1326–1331. 10.1172/JCI117091 8132774 PMC294097

[B28] Hagenbuch B. Scharschmidt B. F. Meier P. J. (1996). Effect of Antisense Oligonucleotides on the Expression of Hepatocellular Bile Acid and Organic Anion Uptake Systems in *Xenopus laevis* Oocytes. Biochem. J. 316 (Pt 3), 901–904. 10.1042/bj3160901 8670169 PMC1217435

[B29] Hu N.-J. Iwata S. Cameron A. D. Drew D. (2011). Crystal Structure of a Bacterial Homologue of the Bile Acid Sodium Symporter ASBT. Nature 478 (7369), 408–411. 10.1038/nature10450 21976025 PMC3198845

[B30] Hu H.-H. Liu J. Lin Y.-L. Luo W.-S. Chu Y.-J. Chang C.-L. (2016). The Rs2296651 (S267F) Variant on NTCP (SLC10A1) Is Inversely Associated with Chronic Hepatitis B and Progression to Cirrhosis and Hepatocellular Carcinoma in Patients with Chronic Hepatitis B. Gut 65 (9), 1514–1521. 10.1136/gutjnl-2015-310686 26642861

[B31] Huang H.-C. Tao M.-H. Hung T.-M. Chen J.-C. Lin Z.-J. Huang C. (2014). (−)-Epigallocatechin-3-gallate Inhibits Entry of Hepatitis B Virus into Hepatocytes. Antiviral Res. 111, 100–111. 10.1016/j.antiviral.2014.09.009 25260897

[B32] Iannacone M. Guidotti L. G. (2022). Immunobiology and Pathogenesis of Hepatitis B Virus Infection. Nat. Rev. Immunol. 22 (1), 19–32. 10.1038/s41577-021-00549-4 34002067

[B33] Inagaki T. Choi M. Moschetta A. Peng L. Cummins C. L. McDonald J. G. (2005). Fibroblast Growth Factor 15 Functions as an Enterohepatic Signal to Regulate Bile Acid Homeostasis. Cel Metab. 2 (4), 217–225. 10.1016/j.cmet.2005.09.001 16213224

[B34] Ito K. Okumura A. Takeuchi J. S. Watashi K. Inoue R. Yamauchi T. (2021). Dual Agonist of Farnesoid X Receptor and Takeda G Protein‐Coupled Receptor 5 Inhibits Hepatitis B Virus Infection *In Vitro* and *In Vivo* . Hepatology 74 (1), 83–98. 10.1002/hep.31712 33434356

[B35] Iwamoto M. Watashi K. Tsukuda S. Aly H. H. Fukasawa M. Fujimoto A. (2014). Evaluation and Identification of Hepatitis B Virus Entry Inhibitors Using HepG2 Cells Overexpressing a Membrane Transporter NTCP. Biochem. Biophys. Res. Commun. 443 (3), 808–813. 10.1016/j.bbrc.2013.12.052 24342612

[B36] Iwamoto M. Saso W. Sugiyama R. Ishii K. Ohki M. Nagamori S. (2019). Epidermal Growth Factor Receptor Is a Host-Entry Cofactor Triggering Hepatitis B Virus Internalization. Proc. Natl. Acad. Sci. U.S.A. 116 (17), 8487–8492. 10.1073/pnas.1811064116 30952782 PMC6486715

[B37] Jung D. Hagenbuch B. Fried M. Meier P. J. Kullak-Ublick G. A. (2004). Role of Liver-Enriched Transcription Factors and Nuclear Receptors in Regulating the Human, Mouse, and Rat NTCP Gene. Am. J. Physiol. Gastrointestinal Liver Physiol. 286 (5), G752–G761. 10.1152/ajpgi.00456.2003 14701722

[B38] Kaneko M. Watashi K. Kamisuki S. Matsunaga H. Iwamoto M. Kawai F. (2015). A Novel Tricyclic Polyketide, Vanitaracin A, Specifically Inhibits the Entry of Hepatitis B and D Viruses by Targeting Sodium Taurocholate Cotransporting Polypeptide. J. Virol. 89 (23), 11945–11953. 10.1128/JVI.01855-15 26378168 PMC4645323

[B39] Kaneko M. Futamura Y. Tsukuda S. Kondoh Y. Sekine T. Hirano H. (2018). Chemical Array System, a Platform to Identify Novel Hepatitis B Virus Entry Inhibitors Targeting Sodium Taurocholate Cotransporting Polypeptide. Sci. Rep. 8 (1), 2769. 10.1038/s41598-018-20987-w 29426822 PMC5807303

[B40] Keitel V. Burdelski M. Warskulat U. Kühlkamp T. Keppler D. Häussinger D. (2005). Expression and Localization of Hepatobiliary Transport Proteins in Progressive Familial Intrahepatic Cholestasis. Hepatology 41 (5), 1160–1172. 10.1002/hep.20682 15841457

[B41] Ko C. Park W.-J. Park S. Kim S. Windisch M. P. Ryu W.-S. (2015). The FDA Approved Drug Irbesartan Inhibits HBV-Infection in HepG2 Cells Stably Expressing Sodium Taurocholate Co-transporting Polypeptide. Antivir. Ther. 20 (8), 835–842. 10.3851/IMP2965 25929767

[B42] König A. Döring B. Mohr C. Geipel A. Geyer J. Glebe D. (2014). Kinetics of the Bile Acid Transporter and Hepatitis B Virus Receptor Na+/taurocholate Cotransporting Polypeptide (NTCP) in Hepatocytes. J. Hepatol. 61 (4), 867–875. 10.1016/j.jhep.2014.05.018 24845614

[B43] Kühlkamp T. Keitel V. Helmer A. Häussinger D. Kubitz R. (2005). Degradation of the Sodium Taurocholate Cotransporting Polypeptide (NTCP) by the Ubiquitin-Proteasome System. Biol. Chem. 386 (10), 1065–1074. 10.1515/BC.2005.122 16218878

[B44] Le Vee M. Gripon P. Stieger B. Fardel O. (2008). Down-Regulation of Organic Anion Transporter Expression in Human Hepatocytes Exposed to the Proinflammatory Cytokine Interleukin 1β. Drug Metab. Dispos. 36 (2), 217–222. 10.1124/dmd.107.016907 17991769

[B45] Le Vee M. Lecureur V. Moreau A. Stieger B. Fardel O. (2009). Differential Regulation of Drug Transporter Expression by Hepatocyte Growth Factor in Primary Human Hepatocytes. Drug Metab. Dispos. 37 (11), 2228–2235. 10.1124/dmd.109.028035 19661216

[B46] Le Vee M. Jouan E. Stieger B. Lecureur V. Fardel O. (2011). Regulation of Drug Transporter Expression by Oncostatin M in Human Hepatocytes. Biochem. Pharmacol. 82 (3), 304–311. 10.1016/j.bcp.2011.04.017 21570956

[B47] Lee H. W. Park H. J. Jin B. Dezhbord M. Kim D. Y. Han K.-H. (2017). Effect of S267F Variant of NTCP on the Patients with Chronic Hepatitis B. Sci. Rep. 7 (1), 17634. 10.1038/s41598-017-17959-x 29247233 PMC5732244

[B48] Lempp F. A. Wiedtke E. Qu B. Roques P. Chemin I. Vondran F. W. R. (2017). Sodium Taurocholate Cotransporting Polypeptide Is the Limiting Host Factor of Hepatitis B Virus Infection in Macaque and Pig Hepatocytes. Hepatology 66 (3), 703–716. 10.1002/hep.29112 28195359

[B49] Li J. Tong S. (2015). From DCPD to NTCP: the Long Journey towards Identifying a Functional Hepatitis B Virus Receptor. Clin. Mol. Hepatol. 21 (3), 193–199. 10.3350/cmh.2015.21.3.193 26523264 PMC4612279

[B50] Li D. Zimmerman T. L. Thevananther S. Lee H.-Y. Kurie J. M. Karpen S. J. (2002). Interleukin-1β-mediated Suppression of RXR:RAR Transactivation of the Ntcp Promoter is JNK-dependent. J. Biol. Chem. 277 (35), 31416–31422. 10.1074/jbc.M204818200 12105223

[B51] Li D. He W. Liu X. Zheng S. Qi Y. Li H. (2017). A Potent Human Neutralizing Antibody Fc-Dependently Reduces Established HBV Infections. Elife 6, e26738. 10.7554/eLife.26738 28949917 PMC5614562

[B52] Liu Y. Zhang L. Yan H. Wang Z. Sun G. Song X. (2021). Design of Dimeric Bile Acid Derivatives as Potent and Selective Human NTCP Inhibitors. J. Med. Chem. 64 (9), 5973–6007. 10.1021/acs.jmedchem.1c00078 33906348

[B53] Lu X. Liu L. Shan W. Kong L. Chen N. Lou Y. (2019). The Role of the Sodium-Taurocholate Co-transporting Polypeptide (NTCP) and Bile Salt Export Pump (BSEP) in Related Liver Disease. Curr. Drug Metab. 20 (5), 377–389. 10.2174/1389200220666190426152830 31258056

[B54] Mita S. Suzuki H. Akita H. Hayashi H. Onuki R. Hofmann A. F. (2006). Vectorial Transport of Unconjugated and Conjugated Bile Salts by Monolayers of LLC-PK1 Cells Doubly Transfected with Human NTCP and BSEP or with Rat Ntcp and Bsep. Am. J. Physiol. Gastrointestinal Liver Physiol. 290 (3), G550–G556. 10.1152/ajpgi.00364.2005 16474011

[B55] Mukhopadhayay S. Ananthanarayanan M. Stieger B. Meier P. J. Suchy F. J. Anwer M. S. (1997). cAMP Increases Liver Na+-Taurocholate Cotransport by Translocating Transporter to Plasma Membranes. Am. J. Physiol. Gastrointestinal Liver Physiol. 273 (4), G842–G848. 10.1152/ajpgi.1997.273.4.G842 9357825

[B56] Müller S. F. König A. Döring B. Glebe D. Geyer J. (2018). Characterisation of the Hepatitis B Virus Cross-Species Transmission Pattern via Na+/taurocholate Co-transporting Polypeptides from 11 New World and Old World Primate Species. PLoS One 13 (6), e0199200. 10.1371/journal.pone.0199200 29912972 PMC6005513

[B57] Nguyen M. H. Wong G. Gane E. Kao J.-H. Dusheiko G. (2020). Hepatitis B Virus: Advances in Prevention, Diagnosis, and Therapy. Clin. Microbiol. Rev. 33 (2), e00046. 10.1128/CMR.00046-19 32102898 PMC7048015

[B58] Ni Y. Lempp F. A. Mehrle S. Nkongolo S. Kaufman C. Fälth M. (2014). Hepatitis B and D Viruses Exploit Sodium Taurocholate Co-transporting Polypeptide for Species-specific Entry into Hepatocytes. Gastroenterology 146 (4), 1070–1083. 10.1053/j.gastro.2013.12.024 24361467

[B59] Nio Y. Akahori Y. Okamura H. Watashi K. Wakita T. Hijikata M. (2018). Inhibitory Effect of Fasiglifam on Hepatitis B Virus Infections through Suppression of the Sodium Taurocholate Cotransporting Polypeptide. Biochem. Biophys. Res. Commun. 501 (3), 820–825. 10.1016/j.bbrc.2018.04.199 29723527

[B60] Nkongolo S. Ni Y. Lempp F. A. Kaufman C. Lindner T. Esser-Nobis K. (2014). Cyclosporin A Inhibits Hepatitis B and Hepatitis D Virus Entry by Cyclophilin-independent Interference with the NTCP Receptor. J. Hepatol. 60 (4), 723–731. 10.1016/j.jhep.2013.11.022 24295872

[B61] Odom D. T. Zizlsperger N. Gordon D. B. Bell G. W. Rinaldi N. J. Murray H. L. (2004). Control of Pancreas and Liver Gene Expression by HNF Transcription Factors. Science 303 (5662), 1378–1381. 10.1126/science.1089769 14988562 PMC3012624

[B62] Park S. W. Schonhoff C. M. Webster C. R. L. Anwer M. S. (2012). Protein Kinase Cδ Differentially Regulates cAMP-dependent Translocation of NTCP and MRP2 to the Plasma Membrane. Am. J. Physiol. Gastrointestinal Liver Physiol. 303 (5), G657–G665. 10.1152/ajpgi.00529.2011 PMC3468552 22744337

[B63] Passioura T. Watashi K. Fukano K. Shimura S. Saso W. Morishita R. (2018). De Novo Macrocyclic Peptide Inhibitors of Hepatitis B Virus Cellular Entry. Cel Chem. Biol. 25 (7), 906–915. 10.1016/j.chembiol.2018.04.011 29779957

[B64] Peng L. Zhao Q. Li Q. Li M. Li C. Xu T. (2015). The p.Ser267Phe Variant inSLC10A1is Associated with Resistance to Chronic Hepatitis B. Hepatology 61 (4), 1251–1260. 10.1002/hep.27608 25418280

[B65] Petzinger E. (1994). Transport of Organic Anions in the Liver. An Update on Bile Acid, Fatty Acid, Monocarboxylate, Anionic Amino Acid, Cholephilic Organic Anion, and Anionic Drug Transport. Rev. Physiol. Biochem. Pharmacol. 123, 47–211. 10.1007/BFb0030903 8209137

[B66] Purushotham A. Xu Q. Lu J. Foley J. F. Yan X. Kim D.-H. (2012). Hepatic Deletion of SIRT1 Decreases Hepatocyte Nuclear Factor 1α/Farnesoid X Receptor Signaling and Induces Formation of Cholesterol Gallstones in Mice. Mol. Cel Biol. 32 (7), 1226–1236. 10.1128/MCB.05988-11 PMC3302441 22290433

[B67] Qiu X. Bi Y.-A. Balogh L. M. Lai Y. (2013). Absolute Measurement of Species Differences in Sodium Taurocholate Cotransporting Polypeptide (NTCP/Ntcp) and its Modulation in Cultured Hepatocytes. J. Pharm. Sci. 102 (9), 3252–3263. 10.1002/jps.23582 23657999

[B68] Ringehan M. McKeating J. A. Protzer U. (2017). Viral Hepatitis and Liver Cancer. Phil. Trans. R. Soc. B 372, 20160274. 10.1098/rstb.2016.0274 28893941 PMC5597741

[B69] Rose A. J. Díaz M. B. Reimann A. Klement J. Walcher T. Krones-Herzig A. (2011). Molecular Control of Systemic Bile Acid Homeostasis by the Liver Glucocorticoid Receptor. Cel Metab. 14 (1), 123–130. 10.1016/j.cmet.2011.04.010 21723510

[B70] Rybicka M. Bielawski K. P. (2020). Recent Advances in Understanding, Diagnosing, and Treating Hepatitis B Virus Infection. Microorganisms 8 (9), 1416. 10.3390/microorganisms8091416 PMC7565763 32942584

[B71] Sarkar S. Bananis E. Nath S. Anwer M. S. Wolkoff A. W. Murray J. W. (2006). PKCζ is Required for Microtubule-Based Motility of Vesicles Containing the Ntcp Transporter. Traffic 7 (8), 1078–1091. 10.1111/j.1600-0854.2006.00447.x 16734659

[B72] Schweitzer A. Horn J. Mikolajczyk R. T. Krause G. Ott J. J. (2015). Estimations of Worldwide Prevalence of Chronic Hepatitis B Virus Infection: a Systematic Review of Data Published between 1965 and 2013. Lancet 386 (10003), 1546–1555. 10.1016/S0140-6736(15)61412-X 26231459

[B73] Shepard C. W. Finelli L. Alter M. J. (2005). Global Epidemiology of Hepatitis C Virus Infection. Lancet Infect. Dis. 5 (9), 558–567. 10.1016/S1473-3099(05)70216-4 16122679

[B74] Shih D. Q. Bussen M. Sehayek E. Ananthanarayanan M. Shneider B. L. Suchy F. J. (2001). Hepatocyte Nuclear Factor-1α Is an Essential Regulator of Bile Acid and Plasma Cholesterol Metabolism. Nat. Genet. 27 (4), 375–382. 10.1038/86871 11279518

[B75] Shimura S. Watashi K. Fukano K. Peel M. Sluder A. Kawai F. (2017). Cyclosporin Derivatives Inhibit Hepatitis B Virus Entry without Interfering with NTCP Transporter Activity. J. Hepatol. 66 (4), 685–692. 10.1016/j.jhep.2016.11.009 27890789 PMC7172969

[B76] Siewert E. Dietrich C. G. Lammert F. Heinrich P. C. Matern S. Gartung C. (2004). Interleukin-6 Regulates Hepatic Transporters during Acute-phase Response. Biochem. Biophys. Res. Commun. 322 (1), 232–238. 10.1016/j.bbrc.2004.07.102 15313196

[B77] Simon F. R. Fortune J. Iwahashi M. Qadri I. Sutherland E. (2004). Multihormonal Regulation of Hepatic sinusoidalNtcpgene Expression. Am. J. Physiol. Gastrointestinal Liver Physiol. 287 (4), G782–G794. 10.1152/ajpgi.00379.2003 15361361

[B78] Slijepcevic D. Roscam Abbing R. L. P. Katafuchi T. Blank A. Donkers J. M. van Hoppe S. (2017). Hepatic Uptake of Conjugated Bile Acids Is Mediated by Both Sodium Taurocholate Cotransporting Polypeptide and Organic Anion Transporting Polypeptides and Modulated by Intestinal Sensing of Plasma Bile Acid Levels in Mice. Hepatology 66 (5), 1631–1643. 10.1002/hep.29251 28498614 PMC5698707

[B79] Sokol R. J. Devereaux M. Dahl R. Gumpricht E. (2006). "Let There Be Bile"-Understanding Hepatic Injury in Cholestasis. J. Pediatr. Gastroenterol. Nutr. 43 (Suppl. 1), S4–S9. 10.1097/01.mpg.0000226384.71859.16 16819400

[B80] Stieger B. (2011). The Role of the Sodium-Taurocholate Cotransporting Polypeptide (NTCP) and of the Bile Salt export Pump (BSEP) in Physiology and Pathophysiology of Bile Formation. Handb Exp. Pharmacol. (201), 205–259. 10.1007/978-3-642-14541-4_5 21103971

[B81] Sureau C. Salisse J. (2013). A Conformational Heparan Sulfate Binding Site Essential to Infectivity Overlaps with the Conserved Hepatitis B Virus A-Determinant. Hepatology 57 (3), 985–994. 10.1002/hep.26125 23161433

[B82] Sureau C. Guerra B. Lanford R. E. (1993). Role of the Large Hepatitis B Virus Envelope Protein in Infectivity of the Hepatitis delta Virion. J. Virol. 67 (1), 366–372. 10.1128/JVI.67.1.366-372.1993 8416375 PMC237372

[B83] Takemori T. Sugimoto-Ishige A. Nishitsuji H. Futamura Y. Harada M. Kimura-Someya T. (2022). Establishment of a Monoclonal Antibody against Human NTCP that Blocks Hepatitis B Virus Infection. J. Virol. 96, JVI0168621. 10.1128/JVI.01686-21 PMC8906425 34985994

[B84] Takeuchi J. S. Fukano K. Iwamoto M. Tsukuda S. Suzuki R. Aizaki H. (2019). A Single Adaptive Mutation in Sodium Taurocholate Cotransporting Polypeptide Induced by Hepadnaviruses Determines Virus Species Specificity. J. Virol. 93 (5), e01432. 10.1128/JVI.01432-18 30541857 PMC6384088

[B85] Ticho A. L. Malhotra P. Dudeja P. K. Gill R. K. Alrefai W. A. (2019). Intestinal Absorption of Bile Acids in Health and Disease. Compr. Physiol. 10 (1), 21–56. 10.1002/cphy.c190007 31853951 PMC7171925

[B86] Trépo C. Chan H. L. Y. Lok A. (2014). Hepatitis B Virus Infection. Lancet 384 (9959), 2053–2063. 10.1016/S0140-6736(14)60220-8 24954675

[B87] Tsukuda S. Watashi K. Iwamoto M. Suzuki R. Aizaki H. Okada M. (2015). Dysregulation of Retinoic Acid Receptor Diminishes Hepatocyte Permissiveness to Hepatitis B Virus Infection through Modulation of Sodium Taurocholate Cotransporting Polypeptide (NTCP) Expression. J. Biol. Chem. 290 (9), 5673–5684. 10.1074/jbc.M114.602540 25550158 PMC4342479

[B88] Tsukuda S. Watashi K. Hojima T. Isogawa M. Iwamoto M. Omagari K. (2017). A New Class of Hepatitis B and D Virus Entry Inhibitors, Proanthocyanidin and its Analogs, that Directly Act on the Viral Large Surface Proteins. Hepatology 65 (4), 1104–1116. 10.1002/hep.28952 27863453

[B89] Uchida T. Park S. B. Inuzuka T. Zhang M. Allen J. N. Chayama K. (2021). Genetically Edited Hepatic Cells Expressing the NTCP-S267F Variant Are Resistant to Hepatitis B Virus Infection. Mol. Ther. Methods Clin. Develop. 23, 597–605. 10.1016/j.omtm.2021.11.002 PMC8608598 34853804

[B90] Urban S. Bartenschlager R. Kubitz R. Zoulim F. (2014). Strategies to Inhibit Entry of HBV and HDV into Hepatocytes. Gastroenterology 147 (1), 48–64. 10.1053/j.gastro.2014.04.030 24768844

[B91] Vaz F. M. Paulusma C. C. Huidekoper H. de Ru M. Lim C. Koster J. (2015). Sodium Taurocholate Cotransporting Polypeptide (SLC10A1) Deficiency: Conjugated Hypercholanemia without a clear Clinical Phenotype. Hepatology 61 (1), 260–267. 10.1002/hep.27240 24867799

[B92] Visser W. E. Wong W. S. van Mullem A. A. A. Friesema E. C. H. Geyer J. Visser T. J. (2010). Study of the Transport of Thyroid Hormone by Transporters of the SLC10 Family. Mol. Cell Endocrinol. 315 (1-2), 138–145. 10.1016/j.mce.2009.08.003 19682536

[B93] Volz T. Allweiss L. ḾBarek M. B. Warlich M. Lohse A. W. Pollok J. M. (2013). The Entry Inhibitor Myrcludex-B Efficiently Blocks Intrahepatic Virus Spreading in Humanized Mice Previously Infected with Hepatitis B Virus. J. Hepatol. 58 (5), 861–867. 10.1016/j.jhep.2012.12.008 23246506

[B94] Wang L. Han Y. Kim C.-S. Lee Y.-K. Moore D. D. (2003). Resistance of SHP-Null Mice to Bile Acid-Induced Liver Damage. J. Biol. Chem. 278 (45), 44475–44481. 10.1074/jbc.M305258200 12933814

[B95] Watashi K. Wakita T. (2015). Hepatitis B Virus and Hepatitis D Virus Entry, Species Specificity, and Tissue Tropism. Cold Spring Harb Perspect. Med. 5 (8), a021378. 10.1101/cshperspect.a021378 26238794 PMC4526719

[B96] Watashi K. Urban S. Li W. Wakita T. (2014). NTCP and beyond: Opening the Door to Unveil Hepatitis B Virus Entry. Int. J. Mol. Sci. 15 (2), 2892–2905. 10.3390/ijms15022892 24557582 PMC3958888

[B97] Watashi K. Sluder A. Daito T. Matsunaga S. Ryo A. Nagamori S. (2014). Cyclosporin A and its Analogs Inhibit Hepatitis B Virus Entry into Cultured Hepatocytes through Targeting a Membrane Transporter, Sodium Taurocholate Cotransporting Polypeptide (NTCP). Hepatology 59 (5), 1726–1737. 10.1002/hep.26982 24375637 PMC4265264

[B98] Webster C. R. L. Anwer M. S. (1999). Role of the PI3K/PKB Signaling Pathway in cAMP-Mediated Translocation of Rat Liver Ntcp. Am. J. Physiol. Gastrointestinal Liver Physiol. 277 (6), G1165–G1172. 10.1152/ajpgi.1999.277.6.G1165 10600813

[B99] Webster C. R. L. Blanch C. J. Phillips J. Anwer M. S. (2000). Cell Swelling-Induced Translocation of Rat Liver Na+/Taurocholate Cotransport Polypeptide Is Mediated via the Phosphoinositide 3-Kinase Signaling Pathway. J. Biol. Chem. 275 (38), 29754–29760. 10.1074/jbc.M002831200 10889198

[B100] Webster C. R. L. Srinivasulu U. Ananthanarayanan M. Suchy F. J. Anwer M. S. (2002). Protein Kinase B/Akt Mediates cAMP- and Cell Swelling-Stimulated Na+/taurocholate Cotransport and Ntcp Translocation. J. Biol. Chem. 277 (32), 28578–28583. 10.1074/jbc.M201937200 12034724

[B101] Xia Y. Carpentier A. Cheng X. Block P. D. Zhao Y. Zhang Z. (2017). Human Stem Cell-Derived Hepatocytes as a Model for Hepatitis B Virus Infection, Spreading and Virus-Host Interactions. J. Hepatol. 66 (3), 494–503. 10.1016/j.jhep.2016.10.009 27746336 PMC5316493

[B102] Xiao Y. Liu C. Tang W. Zhang H. Chen X. (2019). Evans Blue Inhibits HBV Replication through a Dual Antiviral Mechanism by Targeting Virus Binding and Capsid Assembly. Front. Microbiol. 10, 2638. 10.3389/fmicb.2019.02638 31798562 PMC6868041

[B103] Yan H. Zhong G. Xu G. He W. Jing Z. Gao Z. (2012). Sodium Taurocholate Cotransporting Polypeptide Is a Functional Receptor for Human Hepatitis B and D Virus. Elife 1, e00049. 10.7554/eLife.00049 23150796 PMC3485615

[B104] Yan H. Peng B. He W. Zhong G. Qi Y. Ren B. (2013). Molecular Determinants of Hepatitis B and D Virus Entry Restriction in Mouse Sodium Taurocholate Cotransporting Polypeptide. J. Virol. 87 (14), 7977–7991. 10.1128/JVI.03540-12 23678176 PMC3700185

[B105] Yan H. Peng B. Liu Y. Xu G. He W. Ren B. (2014). Viral Entry of Hepatitis B and D Viruses and Bile Salts Transportation Share Common Molecular Determinants on Sodium Taurocholate Cotransporting Polypeptide. J. Virol. 88 (6), 3273–3284. 10.1128/JVI.03478-13 24390325 PMC3957944

[B106] Yan Y. Allweiss L. Yang D. Kang J. Wang J. Qian X. (2019). Down-regulation of Cell Membrane Localized NTCP Expression in Proliferating Hepatocytes Prevents Hepatitis B Virus Infection. Emerging Microbes Infections 8 (1), 879–894. 10.1080/22221751.2019.1625728 31179847 PMC6567113

[B107] Yu L. Liu X. Li X. Yuan Z. Yang H. Zhang L. (2016). Protective Effects of SRT1720 via the HNF1α/FXR Signalling Pathway and Anti-inflammatory Mechanisms in Mice with Estrogen-Induced Cholestatic Liver Injury. Toxicol. Lett. 264, 1–11. 10.1016/j.toxlet.2016.10.016 27818225

[B108] Yuen M.-F. Heo J. Jang J.-W. Yoon J.-H. Kweon Y.-O. Park S.-J. (2021). Safety, Tolerability and Antiviral Activity of the Antisense Oligonucleotide Bepirovirsen in Patients with Chronic Hepatitis B: a Phase 2 Randomized Controlled Trial. Nat. Med. 27 (10), 1725–1734. 10.1038/s41591-021-01513-4 34642494 PMC8516644

[B109] Zhang J. Fu L.-l. Tian M. Liu H.-q. Li J.-j. Li Y. (2015). Design and Synthesis of a Novel Candidate Compound NTI-007 Targeting Sodium Taurocholate Cotransporting Polypeptide [NTCP]-APOA1-HBx-Beclin1-mediated Autophagic Pathway in HBV Therapy. Bioorg. Med. Chem. 23 (5), 976–984. 10.1016/j.bmc.2015.01.020 25650312

[B110] Zhao P. Yang X. Qi S. Liu H. Jiang H. Hoppmann S. (2013). Molecular Imaging of Hepatocellular Carcinoma Xenografts with Epidermal Growth Factor Receptor Targeted Affibody Probes. Biomed. Res. Int. 2013, 1–11. 10.1155/2013/759057 PMC3654646 23710458

[B111] Zhao X. Iqbal W. Sun P. Zhou X. (2021). Na+-Taurocholate Co-transporting Polypeptide (NTCP) in Livers, Function, Expression Regulation, and Potential in Hepatitis B Treatment. Livers 1 (4), 236–249. 10.3390/livers1040019

[B112] Zollner G. Fickert P. Zenz R. Fuchsbichler A. Stumptner C. Kenner L. (2001). Hepatobiliary Transporter Expression in Percutaneous Liver Biopsies of Patients with Cholestatic Liver Diseases. Hepatology 33 (3), 633–646. 10.1053/jhep.2001.22646 11230744

